# Case report: Suspecting guanine nucleotide-binding protein beta 1 mutation in dyskinetic cerebral palsy is important

**DOI:** 10.3389/fped.2023.1204360

**Published:** 2023-10-13

**Authors:** Han-Byeol Choi, Yoonju Na, Jiwon Lee, Jeehun Lee, Ja-Hyun Jang, Jong-Won Kim, Jeong-Yi Kwon

**Affiliations:** ^1^ Department of Physical and Rehabilitation Medicine, Samsung Medical Center, Sungkyunkwan University School of Medicine, Seoul, Republic of Korea; ^2^ Department of Pediatrics, Samsung Medical Center, Sungkyunkwan University School of Medicine, Seoul, Republic of Korea; ^3^ Department of Laboratory Medicine and Genetics, Samsung Medical Center, Sungkyunkwan University School of Medicine, Seoul, Republic of Korea; ^4^ Department of Health Science and Technology, Samsung Advanced Institute for Health Science and Technology, Sungkyunkwan University, Seoul, Republic of Korea

**Keywords:** cerebral palsy, dystonia, GNB1, developmental delay, case report

## Abstract

Herein, we describe the case of a 43-month-old girl who presented with clinical manifestations of dyskinetic cerebral palsy (CP), classified as the Gross Motor Function Classification System (GMFCS) V. The patient had no family history of neurological or perinatal disorders. Despite early rehabilitation, serial assessments using the Gross Motor Function Measure (GMFM) showed no significant improvements in gross motor function. Brain magnetic resonance imaging showed nonspecific findings that could not account for developmental delay or dystonia. Whole-genome sequencing identified a heterozygous NM_002074.5(GNB1):c.239T>C (p.Ile80Thr) mutation in guanine nucleotide-binding protein beta 1 (*GNB1*) gene. Considering this case and previous studies, genetic testing for the etiology of dyskinetic CP is recommended for children without relevant or with nonspecific brain lesions.

## 1. Introduction

Cerebral palsy (CP) is a group of neurodevelopmental disorders that affects posture and movement due to non-progressive disruptions in the developing brain during the fetal period or infancy (1). Known risk factors for CP include prematurity (2), low birth weight (2), multiple births (3), assisted reproductive technology infertility treatment (4), infections during pregnancy (5), jaundice and kernicterus, maternal medical conditions, and birth hypoxic-ischemic brain injury (6), etc. When these traditional risk factors are absent and brain magnetic resonance imaging (MRI) shows no clear abnormalities, the etiology of CP remains unknown. Recently, cohort-based whole-exome and whole-genome sequencing studies have revealed that genetic mutations are important causes of CP (7).

Guanine nucleotide-binding protein beta 1 encephalopathy (*GNB1*-E) is a genetic disease that can be passed down in an autosomal dominant manner or results from a *de novo* mutation (8). Patients with *GNB1* mutations typically exhibit moderate-to-severe developmental delays or intellectual disabilities, often accompanied with structural abnormalities in the brain and epilepsy (8). The patients also demonstrate generalized hypotonia, which may evolve into hypertonia over time, and movement abnormalities such as dystonia, spasticity, athetoid, and chorea (8, 9). Herein, we report the case of a child with dyskinetic CP who had a *GNB1* mutation, as observed in whole genome sequencing.

## 2. Case report

The patient was a 43-month-old girl who was the only child of a couple with no family history of neurological disorders. She was born at 37 weeks of gestation, weighing 2,300 g, and without perinatal problems.

At 6 months of age, she was referred to the Pediatric Physical and Rehabilitation Medicine Clinic because of axial hypotonia and poor head control. She was unable to support her elbows in the prone position, maintain a sitting position, reach out, or bring objects to the midline. At the age of 6 months, a neurological examination revealed truncal hypotonia, mild rigidity in the lower extremities, positive Babinski reflexes, and a positive asymmetric tonic neck reflex to the right. Gradually, she developed nystagmus and dystonia with mild spasticity in the extremities and was diagnosed with dyskinetic CP at the age of 1 year. Despite early and intensive rehabilitation, the patient's development did not improve as per the expectations of the clinical team.

**Figure 1:**
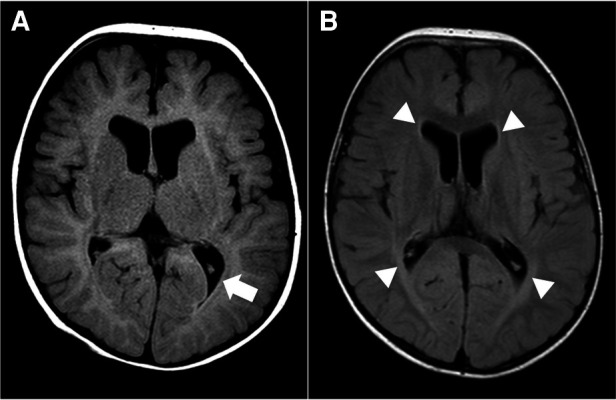
Brain magnetic resonance image (MRI) at 3 months (**A**) and 25 months (**B**) of age. (**A**) Axial T2-fluid attenuated inversion recovery (FLAIR) image of the brain demonstrates periventricular leukomalacia (arrow) with mild ventriculomegaly of the lateral ventricle. (**B**) Axial T2-FLAIR image of the brain shows abnormal high-signal intensity in the white matter, especially in the post-periventricular region and around the frontal horn (arrowhead). There is no interval change in ventriculomegaly of the lateral ventricle.

**Figure 2:**
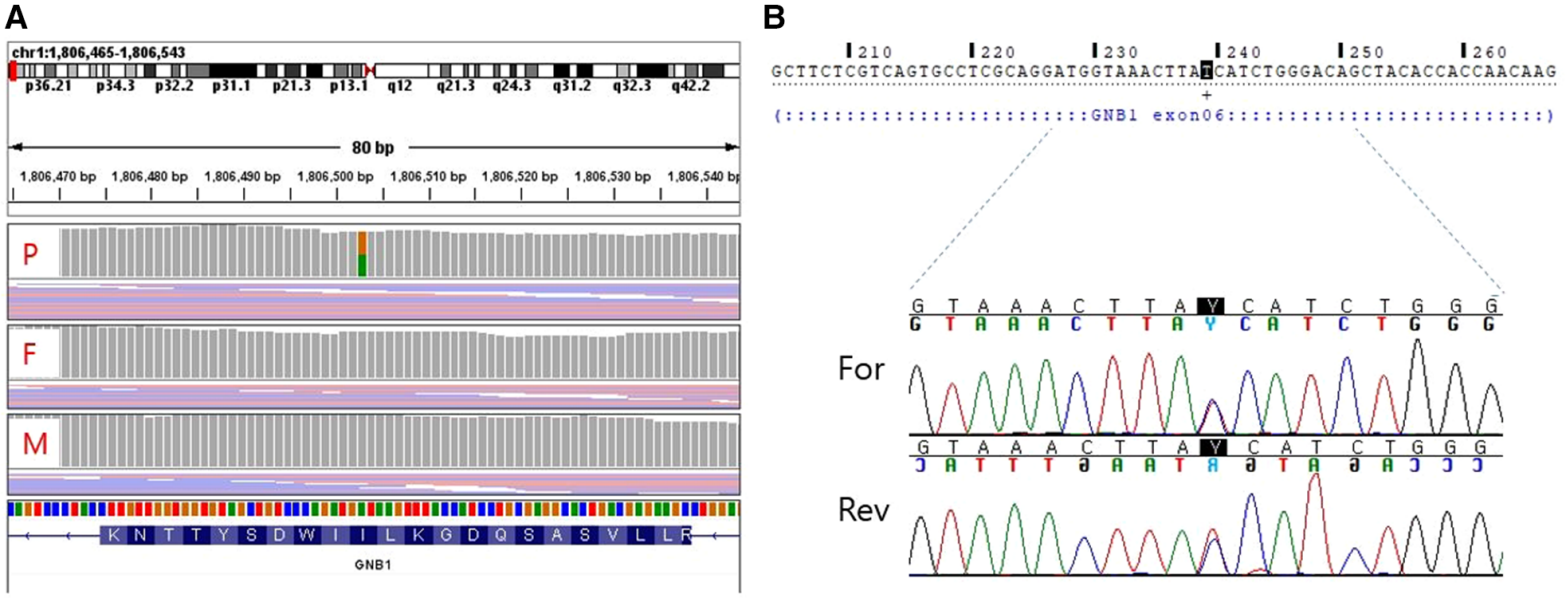
Whole genome sequencing (**A**) and sanger sequencing (**B**) validation of the target mutation [NM_002074.5(GNB1):c.239T > C]. (**A**) A *de novo* variant is detected in the *GNB1* gene of the proband. (**B**) Sanger sequencing shows a heterozygous missense variant of *GNB1.* P, proband; F, father; M, mother; For, Forward; Rev, Reverse.

For diagnostic workup, a brain MRI was performed at the age of 3 months, which suggested periventricular leukomalacia with mild ventriculomegaly (Figure 1A). At the age of 25 months, a follow-up brain MRI showed an apparent abnormal T2-fluid attenuated inversion recovery (FLAIR) high-signal intensity in the white matter, particularly in the post-periventricular region and around the frontal horn, along with cortical atrophy in the frontoparietal lobe (Figure 1B). Subsequently, genetic testing was recommended, and a pathogenic *de novo* mutation in *GNB1* was identified using whole-genome sequencing. A single variant of *GNB1*, NM_002074.5(GNB1):c.239T>C (p.Ile80Thr), was identified through whole-genome sequencing and subsequently confirmed by Sanger sequencing (Figures 2A,B).

**Figure 3:**
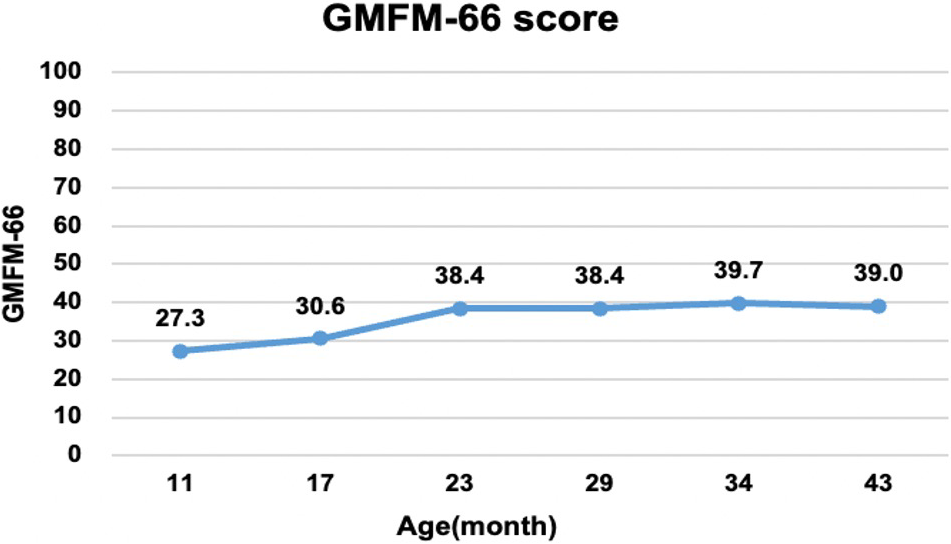
Changes in the gross motor function measure (GMFM-66). The graph shows the total score of the GMFM-66 evaluated at 6–12 months intervals from 11 to 43 months of age. Despite early intensive rehabilitation, she showed poor improvement on the GMFM-66, reaching a plateau at approximately 24 months.

**Figure 4:**
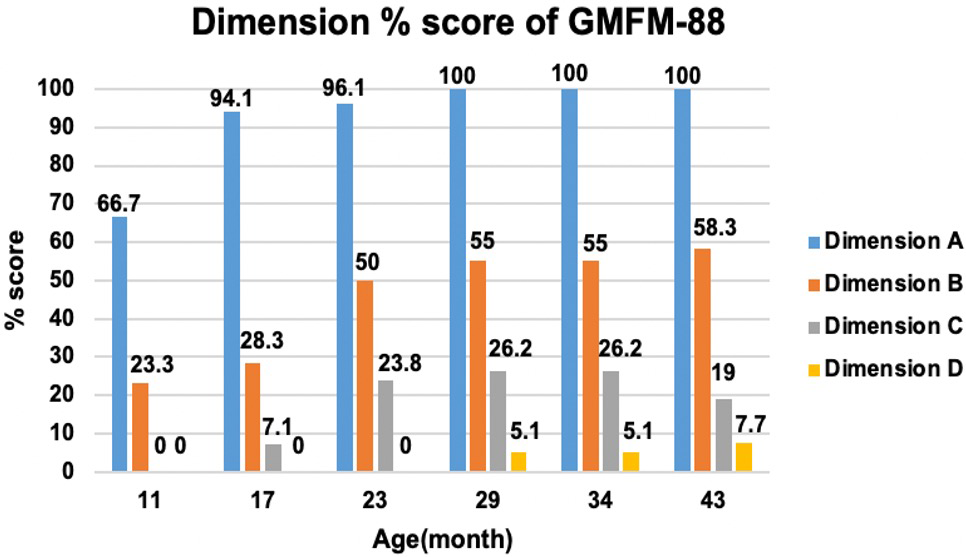
Change in dimension % score of gross motor function measure (GMFM-88). The graph examines the trend of change by subdividing GMFM-88 from dimension A to D. Dimension A stands for lying and rolling, dimension B stands for sitting, dimension C stands for crowing and kneeling, and dimension D stands for standing. She showed poor improvement in dimensions C and D.

An intelligence test was performed at 41 months of age, and her measured IQ was 42 according to the Korean Wechsler Preschool and Primary Scale of Intelligence Fourth Edition (K-WPPSI-IV) (10). Her social maturity score on the Korean Vineland Adaptive Behavior Scale Second Edition (K-Vineland-II) (11) was 44, indicating intellectual disability. Language evaluation conducted at the same age showed that the equivalent ages of receptive and expressive languages were 18 and 8 months, respectively. At 41 months of age, the Korean Bayley Scales of Infant and Toddler Development Third Edition (K-Bayley-III) (12) revealed a significant developmental delay on the cognitive (55, 0.1 percentile), language (49, <0.1 percentile), motor (49, <0.1 percentile), and social-emotional scales (65, 1 percentile). Gross motor function was evaluated using the Gross Motor Function Measure (GMFM-66 & GMFM-88) (13) from the ages of 11 to 43 months; it is currently classified as the Gross Motor Function Classification System (GMFCS) (14) V and Mini-Manual Ability Classification System (Mini-MACS) (15) IV. Changes in GMFM-66 and GMFM-88 scores during this period are shown in Figures 3 and 4, respectively.

The GMFM-66 score was 38.4 at the age of 23 months and reached an early plateau. A similar pattern was observed in dimension B (sitting), C (crowing and kneeling), and D (standing) scores of the GMFM-88. In particular, the scores for dimension C (crowing and kneeling) and D (standing) showed very poor improvement, with only 26.2% and 51.0% respectively at the age of 34 months.

Written informed consent was obtained from the patient’s parents for the publication of any potentially identifiable images and data included in this article.

## 3. Discussion

Herein, we present the case of a child with *GNB1*-E who exhibited significant developmental delay, abnormal muscle tone (initial hypotonia followed by hypertonia), dystonia with mild spasticity, nystagmus, and non-specific brain MRI findings. While this case report presents clinical findings of only one individual, it is clinically valuable as it describes the detailed trajectory of motor developmental delay, which is the most important phenotype of CP resulting from *GNB1*-E. To our knowledge, no previous studies have described the early motor development of patients with CP resulting from *GNB1*-E.

Although premature birth, hypoxic-ischemic encephalopathy, prenatal infection, etc. are established risk factors for CP, approximately one-third of children with CP do not have these typical risk factors (16). Genetic factors are suspected to be the underlying causes in many such children. Until a recent date, it had been suggested that the contribution of genetic variants to the burden of CP was about 2% (17). However, since the introduction of affordable new-generation DNA sequencing, the focus of genetic investigations in CP shifted from gene association studies to the identification of the likely causal variants (18). Several of single-gene causes of CP have been revealed through studies of families with more than two individuals with CP, such as the *KANK1, AP4MI,* and *GAD1* gene mutations (19, 20). Recent investigations of genetic causes in a large cohort of singleton CP cases using Whole Exome Sequencing have identified that the proportion of the cases carrying plausible genetic mutation is much larger than previous reports. At least 14% of nearly 200 singleton cases with CP have been found to have a plausible genetic mutation, *de novo* or inherited (21).

“CP mimics” is a term used to describe a condition that exhibits clinical symptoms consistent with CP, motor impairments appearing in infancy or early childhood, even in the absence of typical risk factors or relevant neuroimaging abnormalities (22). “CP mimics” encompass a spectrum of disorders, including inborn errors of metabolism like Lesch Nyhan disease and Glut1 deficiency, as well as neurogenetic disorders such as Krabbe disease and Metachromatic leukodystrophy (22). However, the diagnostic process for cerebral palsy primarily hinges on the clinical presentations, not the underlying cause including genetic mutations in this case. Consequently, the International Cerebral Palsy Genomics Consortium underscores that when these genetic mutations are discovered in individuals manifesting clinical manifestations of cerebral palsy, they should still be diagnosed as CP itself, rather than categorized as CP mimics (23).

In a large retrospective cohort study of 1,526 patients with CP, 229 genes with pathogenic mutations were identified in 450 patients (23). A total of 86 genes, including *CTNNB1, KIF1A, GNAO1, and TUBA1A,* were mutated in two or more patients, and *GNB1* mutations were identified in two patients. Although the clinical features of these children were not described in the study, the two children with *GNB1* mutation harbored the Ile80Thr variant as a *de novo* mutation. Lewis et al. proposed a set of criteria for identifying CP-associated genes, which included a series of steps from gene discovery and laboratory investigations to clinical applications (7). Recent studies have shown that *GNB1* mutations alter the activity of G protein-gated inwardly rectifying potassium channels, which are mainly expressed in neurons (24).

A total of 58 cases of *GNB1* mutations have been reported, with 12 cases identified as having the Ile80Thr variant (8). While most of the reported cases were identified as *de novo* mutations, three children inherited pathogenic variants from their parents (p. Arg96Leu, p.Thr243Ala). Pathogenic *GNB1* variants cause neurodevelopmental disabilities with global developmental delays. All children with *GNB1* mutations experienced moderate-to-severe developmental delays. Furthermore, nearly 50% of the patients were nonverbal and nonambulatory (9), which was consistent with the findings in our study. Our patient's gross motor function was regularly assessed using the GMFM and no significant improvement or regression has been observed to date. Despite early intensive rehabilitation, her GMFM-66 score plateaued at 23 months of age; she is now classified as having severe quadriplegic CP, GMFCS V, and Mini-MACS IV.

Abnormal muscle tone and various movement disorders have been reported in *GNB1*-E. Hypotonia is the most common presentation, followed by spasticity and dystonia (8, 9); all of these were observed in our case. In the present case, the patient had severe truncal hypotonia during infancy. Over time, she developed dystonia and mild spasticity in her extremities, leading to the diagnosis of dyskinetic CP. It is still uncertain whether the abnormality of muscle tone shown in the patient is a typical finding of the identified variant (p.Ile80Thr). Endo et al. (25) suggested a possible genotype-phenotype correlation between the p.Ile80Thr variant and severe infantile hypotonia that may progress to hypertonia such as spasticity and dystonia. Further studies are necessary to define the phenotype of GNB1 variants.

Kitai et al. (26) reported the etiology of dyskinetic CP based on the clinical course and brain MRI findings in 163 term-born and 136 preterm-born children. The most common causes of dyskinetic CP were found to be hypoxic-ischemic encephalopathy (HIE) in term infants and bilirubin encephalopathy (BE) in preterm infants. On brain MRI, HIE was characterized by T2 high signal intensity in the bilateral thalamus and posterior putamen, and BE by T2 high signal intensity in the bilateral globus pallidus. Eighteen patients had normal brain MRI results, and genetic mutations were identified in eight of these patients. Our patient did not have basal ganglia lesions commonly associated with dyskinetic CP but rather exhibited non-specific white matter lesions on brain MRI. In addition, there was no evidence of hyperbilirubinemia in the results of the laboratory exam.

Based on previous reports and the present case, genetic testing for determining the etiology of dyskinetic CP is recommended for children without relevant or with nonspecific brain lesions whose motor function has reached an early plateau despite early and intensive rehabilitation. Identifying the genetic causes of CP can provide opportunities to understand its underlying pathophysiology and facilitate the development of new interventions targeting specific genetic pathways. In the context of this case report, for detection of the genetic mutation, whole genome sequencing was undertaken as part of the Bio & Medical Technology Development Program of the National Research Foundation. However, it should be noted that whole genome or exome sequencing is a relatively costly procedure, making it impractical for all cases. Consequently, a more judicious approach is suggested. As an initial step, consultation with a genetic counseling team is advisable. Subsequently, although there is no definitive consensus yet, we should selectively perform sequencing tests on the most potentially relevant genes depending on the patient's phenotype. For example, *SPG* and *ARG1* genes for spastic CP, genes including *PDE10A*, *GNB1*, *FOXG1*, *GNAO1*, *SCN1A*, and *SCN8A* for dyskinetic CP, and *SLC2A1*, *ATM*, and *PLP* genes for ataxia CP should be considered (22). If no significant mutations are identified in these tests, considering whole exome or whole genome sequencing as the next step is recommended. This stepwise approach takes into account both clinical and financial factors, optimizing the potential benefits of genetic testing while managing the associated costs.

## Data Availability

The original contributions presented in the study are included in the article/Supplementary Material, further inquiries can be directed to the corresponding author.
